# Domains of professional practice: analysis of publications in the Journal of the Medical Library Association from 2010 to 2019

**DOI:** 10.5195/jmla.2023.1557

**Published:** 2023-04-21

**Authors:** Holly J. Thompson, Jill T. Boruff, Roy Brown, Alexander J. Carroll, John W. Cyrus, Melanie J. Norton, Katherine G. Akers

**Affiliations:** 1 hjthomps@usc.edu, Head, Science and Engineering Library, University of Southern California, Los Angeles, CA, United States.; 2 jill.boruff@mcgill.ca, Co-Editor in Chief, *Journal of the Medical Library Association*, Associate Librarian, Schulich Library of Physical Sciences, Life Sciences, and Engineering, McGill University, Montreal, QC, Canada.; 3 rebrown2@vcu.edu, Associate Professor, Health Sciences Library, Virginia Commonwealth University, Richmond, VA, United States.; 4 alexander.j.carroll@vanderbilt.edu, Associate Director, Stevenson Science and Engineering Library, Vanderbilt University, Nashville, TN, United States.; 5 cyrusjw@vcu.edu, Associate Professor, Health Sciences Library, Virginia Commonwealth University, Richmond, VA, United States.; 6 melanie.norton@yale.edu, Head, Access and Delivery Services, Harvey Cushing/John Hay Whitney Medical Library, Yale University, New Haven, CT, United States.; 7 kgakers@gmail.com, Former Editor in Chief, *Journal of the Medical Library Association*, Senior Research Scientist, Precisionheor, Boston, MA, United States.

**Keywords:** MLA domain hubs, content analysis

## Abstract

The Medical Library Association (MLA) has defined 7 domain hubs aligning to different areas of information professional practice. To assess the extent to which content in the *Journal of the Medical Library Association* (*JMLA*) is reflective of these domains, we analyzed the magnitude of JMLA articles aligning to each domain hub over the last 10 years. Bibliographic records for 453 articles published in *JMLA* from 2010 to 2019 were downloaded from Web of Science and screened using Covidence software. Thirteen articles were excluded during the title and abstract review because they failed to meet the inclusion criteria, resulting in 440 articles included in this review. The title and abstract of each article were screened by two reviewers, each of whom assigned the article up to two tags corresponding to MLA domain hubs (i.e., information services, information management, education, professionalism and leadership, innovation and research practice, clinical support, and health equity & global health). These results inform the MLA community about our strengths in health information professional practice as reflected by articles published in *JMLA*.

## RELATING THE NEW MLA STRUCTURE TO JMLA PUBLICATIONS

In 2018, the Medical Library Association (MLA) announced that it would be restructuring the organization to create member caucuses that would replace the Sections and Special Interest Groups (SIGs). This change was implemented in September 2019 [1].

MLA also announced a plan to establish seven collaborative areas for caucuses, each aligned with a professional practice area, which are called domain hubs [2]. The domain hubs became fully operational in May 2020.

The seven domain hubs and their professional practice areas, as defined by MLA [2], are:

### Clinical Support

Professional practice area covers: evidence-based practice curriculum and habits in health professions, role of librarianship in clinical settings, provision of high-quality health information to consumers.

### Education

Professional practice area covers: pedagogy/andragogy, instruction to health professionals, educational technology, librarian as instructor, instructional design, information literacy.

### Health Equity & Global Health

Professional practice area covers: development of health information professionals globally, equity in access to health information, international collaborations for MLA and medical librarianship.

### Information Management

Professional practice area covers: meta data, representation of information, collection of information, research data management.

### Information Services

Professional practice area covers: research assistance, outreach to specific communities, subject knowledge development, expert searching.

### Innovation & Research Practice

Professional practice area covers: evidence-based librarianship, informatics, research training, diversity in scientific research, assessment, and evaluation.

### Professionalism & Leadership

Professional practice area covers: ethics; equity, diversity, and inclusion; development of leaders; management (human resources, fiscal, project, etc.); influence in health care organizations; education of and advocacy for health information professionals.

In the fall of 2019, a group of Journal of the Medical Library Association (JMLA) Editorial Board members and the Editor-in-Chief came together to map prior *JMLA* publications to the new MLA domain hubs. The team wanted to better understand the distribution of publications related to each domain hub and identify which practice areas, if any, were under-represented.

## CONDUCTING THE ANALYSIS

The Web of Science Core Collection was searched for articles published in *JMLA* between January 2010 and December 2019. Specific publication types were intentionally excluded since the analysis focused on original research. Knowledge syntheses, original investigations, and case reports were included. Proceedings of the annual MLA meeting, lectures and awards, historical topics, brief communications, memoriam columns, tools and skills, resource and book reviews, virtual projects, commentaries, letters to the editor, and editorials were excluded from this analysis. A total of 453 articles were exported for screening using the Covidence application. Thirteen additional articles were excluded from the sample after exporting due to their publication types.

Once the articles were imported into Covidence and before starting the screening process, the research team participated in a calibration exercise using the same 10 articles to develop a consistent application of the tags that would be used to categorize the articles. The available tags were the 7 MLA domain hubs, as defined by MLA and described in the previous section, plus an “ Other ” tag that would indicate articles that did not align with any of the domain hubs. Once the team was calibrated, each of the 440 articles were screened by 2 people who applied up to 2 tags per article to indicate with which domain hub the articles were most closely aligned. The team decided that each article could have no more than 2 tags applied to it. Any conflict, indicated by an article having more than 2 assigned tags, was arbitrated by a third person who would determine which 2 tags were most appropriate for that article. Only 28 articles out of 440 required arbitration.

## INITIAL RESULTS

**Figure 1:**
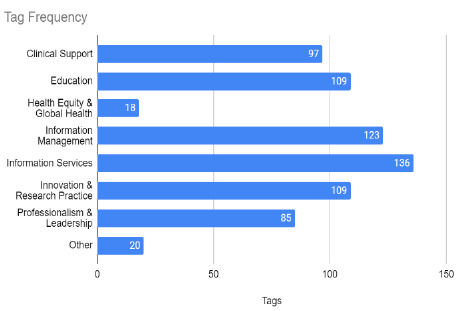
Total tag frequency.

A total of 440 articles were screened by 7 reviewers. Every article was assigned at least 1 tag, and 259 articles were given 2 tags. All domain hub tags were applied to articles at least 85 times, except for Health Equity & Global Health, which was only assigned to 18 articles. Twenty articles were tagged with “Other” and pertained to topics including the history of medicine, access services, and health policy, which are not topics or professional areas of practice included in domain hub definitions. The team also identified a lack of inclusion of some relevant library topics in the MLA domain hub definitions. For example, no domain hub definition explicitly includes collection development, so the team decided to assign any articles relating to collections to the “Information Management” domain hub. Figure 1 illustrates how many times each tag was applied to the articles in the dataset, including articles that received two tags.

The team explored domains that were frequently tagged together to highlight complementary topics and collaborative domain hubs, but recurring combinations were not frequent. For example, the domains most commonly tagged together were Clinical Support and Education, which were jointly applied to a total of 24 articles out of 440.

## SOME CHALLENGES AND ADDITIONAL RESULTS

During the screening process, the research team encountered a wide variety of articles that, for the purposes of this analysis, had to be assigned to at least one domain hub and/or the “Other” category. Some domain hub definitions were so broad that different types of articles were ultimately grouped together. As mentioned above, there were 20 articles that did not fit into any of the domain hub definitions and were categorized simply as “Other.” Table 1 illustrates the range of articles that fall into a selection of the domain hubs and articles that were not so easily categorized.

**Table 1:** Example articles categorized by domain hub.

Information Management
Sugrim S, Schimming L, Halevi G. Identifying e-books authored by faculty: a method for scoping the digital collection and curating a list. 2019.
Rosenzweig M, Schnitzer AE, Song J, Martin S, Ottaviani J. National Institutes of Health public access policy and the University of Michigan Libraries' role in assisting with depositing to PubMed Central. 2011.
Bohle S. “Plutchik”: artificial intelligence chatbot for searching NCBI databases. 2018.
Information Services
Schulte SJ. Eliminating traditional reference services in an academic health sciences library: a case study. 2011.
Hanneke R, O'Brien KK. Comparison of three web-scale discovery services for health sciences research. 2016.
Neilson C, Le ML. A failed attempt at developing a search filter for systematic review methodology articles in Ovid Embase. 2019.
Innovation & Research Practice
Yeh ST, Fontenelle C. Usability study of a mobile website: the Health Sciences Library, University of Colorado Anschutz Medical Campus, experience. 2012.
Sibbald SL, Wathen CN, Kothari A, Day AMB. Knowledge flow and exchange in interdisciplinary primary health care teams (PHCTs): an exploratory study. 2013.
Grinstead C, Schwartz A. Multisite collaboration using REDCap to capture library data. 2019.
Other
Badgett RG, Fernandez JG. Are proposals by politicians for health care reform based on evidence? 2013.
Speaker SL. An historical overview of the National Network of Libraries of Medicine, 1985-2015. 2018.
Jackson A. Reforming the veteran: propaganda and agency in the First World War Reconstruction hospitals. 2019.

The team also explored the most highly cited articles by domain hub to understand which domain hubs have a wider audience or impact. Table 2 lists the top ten most highly cited articles in the study sample, which represent six out of the seven domain hubs. Articles in this list that received two tags had more citations than the articles receiving only one tag. This is possibly attributed to articles with two tags having a wider audience given that their subject matter relates to two professional areas of practice. The domain hub Professionalism & Leadership is not represented in this table nor is the “Other” category. The articles with the highest citations associated with these two categories received forty-four [1] and seventeen [2] citations, respectively. For context, in this study sample, the average article published in JMLA was cited less than once.

**Table 2:** The top ten most highly cited articles in the study sample. Data retrieved from Web of Science “Times Cited, All Databases” September 2022.

Article	Tags	Times Cited
Eriksen MB, Frandsen TF. The impact of patient, intervention, comparison, outcome (PICO) as a search strategy tool on literature search quality: a systematic review. 2018.	Education; Innovation & Research Practice	186
Majid S, Foo S, Luyt B, Zhang X, Theng YL, Chang YK, Mokhtar IA. Adopting evidencebased practice in clinical decision making: nurses' perceptions, knowledge, and barriers. 2011.	Clinical Support; Information Services	164
Boruff JT, Storie D. Mobile devices in medicine: a survey of how medical students, residents, and faculty use smartphones and other mobile devices to find information. 2014.	Clinical Support; Information Management	155
Bramer WM, de Jonge GB, Rethlefsen ML, Mast F, Kleijnen J. A systematic approach to searching: an efficient and complete method to develop literature searches. 2018.	Information Management; Information Services	142
Davis PM, Walters WH. The impact of free access to the scientific literature: a review of recent research. 2011.	Health Equity & Global Health; Information Management	89
Bramer WM, Milic J, Mast F. Reviewing retrieved references for inclusion in systematic reviews using EndNote. 2017.	Information Management; Information Services	80
Marshall JG, Sollenberger J, Easterby-Gannett S, Morgan LK, Klem ML, Cavanaugh SK, Oliver KB, Thompson CA, Romanosky N, Hunter S. The value of library and information services in patient care: results of a multisite study. 2013.	Clinical Support	77
Cooper ID. What is a mapping study?''. 2016.	Innovation & Research Practice	74
Sarli CC, Dubinsky EK, Holmes KL. Beyond citation analysis: a model for assessment of research impact. 2010.	Innovation & Research Practice	73
Cooper ID, Crum JA. New activities and changing roles of health sciences librarians: a systematic review, 1990-2012. 2013.	Information Services	73

## NEXT STEPS

The major outcome of this analysis was identifying that the Health Equity & Global Health domain hub is seriously underrepresented in JMLA publications when compared to other domain hubs. An area of future exploration is to understand why this is the case and investigate whether this is an issue of needing to solicit more articles from this area, if submissions from this area are less likely to be published, or if there are other factors at play. Related to this, JMLA editorial board members will pursue more robust outreach efforts to all MLA domain hubs and caucuses to encourage manuscript submissions and participation in scholarly communication. Ongoing conversations about demystifying the publishing process, overcoming fears or anxiety about writing and publishing, and seeking understanding about reluctance to publish are already underway. Finally, active collaboration between MLA domain hubs and caucuses independent of the JMLA editorial board liaisons is also encouraged as publications with broader subject matter appeal to more readers and potentially increase the impact of the research.

## Data Availability

The data associated with this review are available in the Open Science Framework at DOI 10.17605/OSF.IO/SP8H4.
